# Fruit and Vegetable Intake and Home Nutrition Environment among Low-Income Minority Households with Elementary-Aged Children

**DOI:** 10.3390/nu15081819

**Published:** 2023-04-10

**Authors:** Brittni Naylor Metoyer, Ru-Jye Chuang, MinJae Lee, Christine Markham, Eric L. Brown, Maha Almohamad, Jayna M. Dave, Shreela V. Sharma

**Affiliations:** 1 Department of Epidemiology, Human Genetics and Environmental Sciences, The University of Texas Health Science Center at Houston (UTHealth) School of Public Health, 1200 Pressler Street, Houston, TX 77030, USA; 2 Peter O’Donnell Jr. School of Public Health, University of Texas Southwestern Medical Center (UTSW), 5323 Harry Hines Blvd, Dallas, TX 75390, USA; 3 Department of Health Promotion and Behavioral Sciences, The University of Texas Health Science Center at Houston (UTHealth) School of Public Health, 7000 Fannin Street, Houston, TX 77030, USA; 4 USDA/ARS Children’s Nutrition Research Center, Department of Pediatrics, Baylor College of Medicine, 1100 Bates Ave, Houston, TX 77030, USA

**Keywords:** fruit and vegetable, healthy eating, low-income populations, mealtimes, home nutrition environment

## Abstract

Racial/ethnic and socioeconomic differences were shown to have an influence on child fruit and vegetable intake. This study examined the associations between parent and child fruit and vegetable intake and the home nutrition environment among Hispanic/Latino and African American families. Through a cross-sectional study design, self-reported surveys (*n* = 6074) were obtained from adult–child dyad participants enrolled in Brighter Bites, an evidence-based health promotion program, in the fall of 2018. For every once/day increase in frequency of parent FV intake, there was an increase in child FV intake by 0.701 times/day (CI: 0.650, 0.751, *p* < 0.001) and 0.916 times/day (CI: 0.762, 1.07; *p* < 0.001) among Hispanic/Latinos and African Americans, respectively. In Hispanic/Latino participants, significant positive associations were found between fruits as well as vegetables served at mealtimes ≥3 times/week (*p* < 0.001), family mealtimes 7 times/week (*p* = 0.018), parent–child communication about healthy eating and nutrition at least sometimes during the past 6 months (*p* < 0.05), and frequency of child FV intake, after adjusting for covariates. In African American participants, a significant positive association was found in fruits served at mealtimes ≥1 times/week (*p* < 0.05), and vegetables served at mealtimes ≥5 times/week (*p* < 0.05). Meals cooked from scratch a few times a day/all the time were significantly positively associated with frequency of child FV intake for both Hispanic/Latino (*p* = 0.017) and African American (*p* = 0.007) groups. The relationship between home nutrition environment and child FV intake varied by race and ethnicity. Future programs should consider designing culturally tailored interventions to address racial/ethnic-specific influences that match the child’s race, culture, and ethnicity.

## 1. Introduction

Fruits and vegetables (FV) contain sources of many essential nutrients that are key to a healthy diet [1]. Several studies investigated the relationship between FV consumption improved weight outcomes and disease prevention, along with their nutrient content and subsequent health-promoting effects [2,3,4,5]. In 2017–2018, 21.2% of adolescents (12–19 years) and 20.3% of school-aged children (6–11 years) had the highest prevalence of obesity as compared to 13.4% of preschool-aged children (2–5 years) [6]. Therefore, increased FV consumption was shown to be an effective strategy for weight management and reduced risk of obesity [3,5]. Children with obesity, especially low-income African American children, are at an increased risk for developing type 2 diabetes, cardiovascular disease, hypertension, and asthma [7,8,9,10,11]. Nevertheless, diets rich in FV were shown to reduce the risk of these diet-related health outcomes [12], making FV intake one of the most sought out approaches among childhood disease prevention interventions [13,14,15]. Despite the protective effects of FV [16], many children in the U.S. do not meet the recommended guidelines for FV intake [1,17]. Due to diet-related disparities, minority and low-income children have been at greater risk for not meeting the FV intake recommendations [18,19,20]. 

Parents are known to be nutritional gatekeepers; therefore, they play a key role in establishing a healthy home nutrition environment that would be conducive to improving FV consumption [21]. Positive relationships between parental FV intake [22,23,24], family mealtimes [25,26,27,28,29], home availability of FV [13,27,30,31], and child FV intake were consistently documented in previous literature. Moreover, frequently cooking meals at home was positively associated with a healthier diet [32] and FV intake among adults [33]. To our knowledge, few studies previously explored the association between the frequency of cooked meals from scratch at home and child FV intake. 

Research found racial/ethnic and socioeconomic differences in influences on child FV intake between African American and Hispanic populations [27,30,34,35,36]. Previous studies showed that Hispanic families consume more family meals together [27,36] and have more fresh FV available in the home than African American families [27]. Additionally, a systematic review of the determinants of FV intake in low-income children found a lack of association between home availability of FV and child FV intake among low-income children [37]. Because of the sociodemographic and cultural factors that compound the relationship between the home nutrition environment and child FV intake [38,39], it is essential that racial/ethnic groups be studied separately. A better understanding of racial/ethnic-specific influences may call for future interventions to tailor their messaging and strategies to particular racial/ethnic groups, combatting the one-size-fits-all approaches that were customarily implemented in low-income minority nutrition interventions.

The purpose of this study was to examine the associations between parent FV intake and home nutrition environment and child FV intake among Hispanic/Latino and African American children and parents participating in the Brighter Bites school-based food co-op program in the 2018–2019 school year in the U.S. Brighter Bites, an evidence-based health promotion program, increases access to FV by providing weekly distributions of produce in conjunction with child and parent nutrition education to improve FV intake and the home nutrition environment among low-income families [40,41,42,43]. Examining the associations between parent FV intake and home nutrition environment and child FV intake in African American and Hispanic/Latino families separately may provide important insights to the Brighter Bites program to modify and improve its design, implementation, and evaluation purposes to provide accessible resources effectively to targeted families.

## 2. Materials and Methods

### 2.1. Study Design

We conducted a secondary analysis, re-analyzing existing data to answer a new question of cross-sectional baseline data collected by self-reported parent surveys in the fall of 2018. Families that enrolled by week two of the program implementation received the baseline survey. Enrollment began before the Brighter Bites program started and completed by week two of program implementation for 2018–2019 school year. This study was approved by UTHealth, Committee for Protection of Human Subjects.

### 2.2. Participants

Baseline data from a total of 6074 Hispanic/Latino and African American parent–child dyads participating in Brighter Bites were utilized. The dyads were recruited from public and charter elementary schools, which the degree of regulation varies between the two, in the 6 cities. Informed consent was obtained from the parents who enrolled their family into the Brighter Bites program. There were three main components to its 16-week school-based food co-op program: (1) weekly distributions of fresh produce (~50–60 servings per family) obtained from local food banks and distributors, (2) evidence-based nutrition education through a validated Coordinated Approach to Child Health (CATCH) curriculum that focuses on healthy nutrition and physical activity in schools [44], bilingual nutrition education resources and weekly recipes featuring produce in the distribution bags, and (3) weekly recipe tastings [43]. Participating children and parents of Brighter Bites reported low-income status and mostly identified as Hispanic/Latino (85.1%, 86.2%; respectively) and African American (7.6%, 7.4%; respectively) race/ethnicity. Brighter Bites is currently implemented in 11 cities within the U.S. During the 2018–2019 school year, Brighter Bites was only implemented in 6 cities: Houston, Dallas, Austin, New York City, Washington D.C., and rural area of Southwest Florida.

### 2.3. Data Collection

Voluntary self-reported surveys were administered by paper or electronically, in English and Spanish, to the primary adult caregiver of elementary school age children, 5–12 years of age, in the Brighter Bites participating family that was enrolled by week two of program implementation for the 2018–2019 school year. In total, 6074 self-reported surveys from Hispanic/Latino and African American participating households were collected for this analysis. At least one parent from each participating school completed the survey from Houston, Dallas, Austin, Washington D.C., and Southwest Florida in the 2018–2019 year of Brighter Bites implementation (surveys were not administered to families in New York City due to city-specific regulations). Data were collected for on-going program evaluation, and de-identified data were shared with University of Texas Health Science Center (UTHealth) for further analysis. 

### 2.4. Survey Measures

Child and parent FV intake was assessed using previous questions obtained from the 27-item National Cancer Institute’s 2014 Family Life, Activity, Sun, Health, and Eating Study (FLASHE) dietary screener that assesses frequency of food items consumed [45]. Parents were asked how many times in the past week their child drank 100% pure fruit juice and ate fruit, green salad, or non-fried vegetables, and fried and other kinds of potatoes. Response options included: never, 1–2 times per week, 3–4 times per week, 5–6 times per week, and 7 times per week. In sum, 5 questions were asked and categorized into a composite outcome variable “child FV intake”, with a continuous scale ranging from 0 to 5 times per day (Supplementary Materials, Text S1). Similarly, parents were asked how many times in the past week they ate fruit and vegetables (excluding fruit juice and potatoes). Responses were recoded into a composite exposure variable “parent FV intake”, with a continuous scale ranging from 0 to 2 times per day.

Questions pertaining to the home nutrition environment behavior were adapted from previously validated items [15,40,46,47,48]. Meals cooked from scratch at home were assessed by asking the parent how often the family cooked from scratch, and response options included: never, less than once a month, less than once a week, once a week, a few times a week, once a day, and a few times a day/all the time. 

Fruits and vegetables served at mealtimes were assessed by asking the parent: “During the past 7 days, how many days were fresh/frozen vegetables served at any meals in your home?” and “During the past 7 days, how many days were fresh/canned/dried fruit served at any meals in your home?” Response options included: never, 1–2 times per week, 3–4 times per week, 5–6 times per week, and 7 times per week. Family mealtimes were assessed by asking the parent “During the past 7 days, how many days did you eat any meals together with your child?” with the following response options: never, 1–2 times per week, 3–4 times per week, 5–6 times per week, and 7 times per week.

Parent–child communication about healthy eating and nutrition were assessed by asking the parent how often they had conversations with their child about healthy eating and nutrition in the past 6 months. Response options were: never, rarely, sometimes/about half the time, often, and always. 

Demographic characteristics included respondents’ relationship to child, parent race/ethnicity, child gender, parent age, child age, language spoken at home, household size, parent education level, parent employment status, and enrollment in government assistance programs such as Special Supplemental Nutrition Program for Women, Infants, and Children (WIC), Supplemental Nutrition Assistance Program (SNAP), Double Dollars, Medicaid, Medicare, National School Lunch and/or Breakfast Programs (NSLP), and Children’s Health Insurance Program (CHIP). 

Household food security status was measured using the 2-item Hunger Vital Sign screener [49]. Participants were identified as food insecure if they responded, ‘often true’ or ‘sometimes true’ to either of the following questions: “Within the past 3 months, we worried whether our food would run out before we got money to buy more” or “Within the past 3 months, the food we bought just didn’t last and we didn’t have money to get more”.

### 2.5. Data Analyses

For descriptive purposes, the frequencies, means, and standard deviations (SD) were specified for all variables. Differences by race/ethnicity were tested using the Pearson’s chi-square test for categorical variables and *t*-test for continuous variables. We assessed the cross-sectional associations between parent FV intake (exposure) and child FV intake (outcome) at baseline (prior to participating in Brighter Bites) within each racial/ethnic group using multivariable mixed effects linear regression models that accounted for school-level clustering, where participants (level 1) were nested within school site (level 2). Similarly, the cross-sectional associations of the exposures of frequency of meals cooked from scratch, fruits and vegetables served at mealtimes, family mealtimes, and parent–child communication with the outcome, child FV intake were also evaluated. Potential confounding variables including sociodemographic characteristics were evaluated in each model based on change in estimate (>10%), and were then adjusted in the models. Listwise deletion was used to handle missing data. For exploratory analysis, interaction effects among exposure variables were tested while building multivariable models. Significance was assessed with *p* < 0.05 and 95% confidence interval (CI). All analyses were performed using STATA 15.1 (StataCorp, College Station, TX, USA).

## 3. Results

### 3.1. Participant Demographic Characteristics

Table 1 presents the demographic characteristics of the racial/ethnic minority survey respondents who enrolled in Brighter Bites. The survey was administered to all participating families in the 2018–2019 school year (*n* = 6074). A survey was administered starting prior to the Brighter Bites programming and continued until week two of program implementation. Of these respondents, 5601 (92.2%) identified as Hispanic or Latino and 473 (7.8%) identified as African American or Black (hereafter “African American”). At baseline, the average age of adults (participating parents) and the youngest child were 34.8 years old (±7.8) and 6.5 years old (±2.0), respectively. Most respondents identified as a mother of a participating child (93%), were in Houston, Texas (51.8%), primarily homemakers (53.7%), and received no college education (75.8%). The average household size was 5.1 members (±7.8). The NSLP, Medicaid, and SNAP were the most utilized government assistance programs (59.9%, 74.8%, and 34.2%; respectively). Approximately three-fourths (71.8%) of the respondents reported experiencing food insecurity. 

When we compared demographic characteristics by race/ethnicity, results showed that African American respondents had significantly smaller households (4.7 vs. 5.1; *p* < 0.001), a higher education level (58.6% attended college at least 1 year vs. 21.2%, *p* < 0.001), were employed (61.7% vs. 28.9%; *p* < 0.001), and were slightly older (36.9 years vs. 34.6 years, *p* < 0.001) than Hispanic/Latino respondents. Additionally, a significantly higher percentage of African American respondents reported experiencing food insecurity (76.0% vs. 71.5%, *p* = 0.046) as compared to Hispanic/Latino respondents. Moreover, African American respondents reported greater usage of SNAP (48.2% vs. 33.0%, *p* < 0.001), Medicare (17.3% vs. 5.2%, *p* < 0.001), and NSLP (84.5% vs. 74.0%, *p* < 0.001) than Medicaid (54.3% vs. 60.0%, *p* = 0.018) overall, as compared to Hispanic/Latino respondents.

### 3.2. Reported Child and Parent FV Intake and The Home Nutrition Environment

At baseline, the frequency of child FV intake (times/day) and components of the home nutrition environment were significantly different by race/ethnicity (Table 2). African American children had significantly higher frequency of FV intake than Hispanic/Latino children (2.34 times/day vs. 2.02 times/day, *p* < 0.001). This statistically significant difference in FV intake was not present among African American and Hispanic/Latino parents (1.32 times/day vs. 1.28 times/day, *p* = 0.572, respectively). When comparing home nutrition environment behaviors, African American households served more vegetables at meals (37.8% served 5–7 times/week vs. 25.7%, *p* < 0.001), cooked more often from scratch (35.8% cooked a few times a day/all the time vs. 26.6%, *p* < 0.001), and communicated more about healthy eating and nutrition (38.4% talked all of the time vs. 25.6%, *p* < 0.001); while Hispanic/Latino households served more fruits at meals (25.7% served 5–7 times/week vs. 21.8%, *p* = 0.001) and ate more family meals together (67.8% ate together 7 times/week vs. 55.1%, *p* < 0.001).

### 3.3. Relationship between Frequency of Parent and Child FV Intake

There was a significant positive association found between parent and child dietary FV intakes (Table 3). These results demonstrated that parent FV intake was positively associated with child FV intake such that, for every once/day increase in frequency of parent FV intake, there was an increase in child FV intake by 0.701 times per day (CI: 0.650, 0.751, *p* < 0.001) among the Hispanic/Latino population, after adjusting for participation in SNAP and Medicare, parent age, language spoken at home, child age, city, and food insecurity. Similarly, for every once per day increase in parent dietary FV intake, there was an increase in child dietary FV intake by 0.916 times per day (CI: 0.762, 1.07; *p* < 0.001) among the African American population, after adjusting for SNAP, parent education, food insecurity, and child age. 

A significant interaction effect between SNAP participation and parent FV intake in relation to child FV intake was found only in the Hispanic/Latino population (*p* = 0.020), which may strongly indicate that the association between SNAP participation and child FV intake differed by the frequency of parent FV intake (Supplementary Materials, Table S1, Figure S1). Specifically, among the group with parents that consumed FV ≤ 0.5 times/day, children whose parents participated in SNAP had a lower frequency of FV intake as compared to children whose parents did not participate in SNAP. When parents consumed FV 0.714 times/day or more, SNAP participation had a positive association on the frequency of child FV intake, and this positive association became stronger and statistically significant as the frequency of parent FV intake increased. The association between SNAP participation and child FV intake was the strongest among the parents that consumed FV two times/day (ß = 0.128; CI: 0.055, 2.00; *p* < 0.001). 

### 3.4. Relationship between Home Nutrition Environment and Child FV Intake 

After adjusting for covariates, the results of our study showed a significant positive association between frequency of vegetables and fruits served at mealtimes ≥3 times/week and child FV intake (*p* < 0.001) among Hispanic/Latinos (Table 3). In addition, frequency of family mealtimes 7 times/week, communication about health, and meals cooked from scratch a few times a day/all the time was significantly associated with child FV intake (*p* = 0.018, *p* < 0.05, *p* = 0.017; respectively). In African American families, the same significant positive association was found in vegetables served at mealtimes ≥5 times/week (*p* < 0.05), fruits served at mealtimes ≥1 times/week (*p* < 0.05), and meals cooked from scratch a few times a day/all the time (*p* = 0.007), after adjusting for covariates. There were no significant associations between family mealtimes and parent–child communication about healthy eating and nutrition and child FV intake among African American households (*p* > 0.05). 

## 4. Discussion

The aim of this study was to examine the cross-sectional associations between baseline parent FV intake, home nutrition environment, and child FV intake among low-income minority Brighter Bites participants from the 2018–2019 school year. Our results revealed an overall positive association between the frequency of parent and child FV intake. These findings strengthened the existing literature in this area that demonstrated this positive association and the relevance of potential parental influence on child dietary intake [22,23,24]. 

The frequency of FV served at mealtimes was overall positively associated with higher frequency of FV intake among Hispanic/Latino children. These findings, and other studies among this population, demonstrated a greater likelihood for Hispanic/Latino children to consume FV if they were more frequently served at mealtimes [27,50,51,52]. Similarly, the same positive association was found among African American households in this study, with FV intake being the highest among children that were served FV at least five times/week. These findings accentuate the need to further focus research on effective strategies to increase the frequency of FV being served at mealtimes as a means to improve child FV intake. 

In addition, our study found cooking meals from scratch at home a few times per day/all the time to be significantly associated with increased frequency of child FV intake in both ethnic groups. This finding was consistent with previous studies that showed cooking more frequent meals from scratch to be positively associated with higher FV intake [33,53,54]. Furthermore, cooking from scratch at home less than a few times/week was negatively associated with child FV intake among Hispanic/Latino households. Conversely, this association was not seen in African American households. Although our results indicated that all children would benefit from more frequent produce served at mealtimes and cooked meals from scratch, fewer households in our study reported partaking in these home nutrition environment behaviors consistently. For many low-income minority families, accessibility of produce, cost, time constraints, and lack of cooking skills are barriers to the ability to serve more produce at meals and cook more meals from scratch at home [55,56]. Thus, interventions and initiatives that provide access to produce, and improve food-related skills (e.g., following recipes, meal planning, shopping with a grocery list) and cooking skills, are critical in overcoming these barriers and increasing child FV intake [57,58].

Furthermore, we found significantly higher FV intake among Hispanic/Latino children who ate meals with their family seven times/week. Conversely, there were no significant associations found among African American respondents. Our findings contradicted existing studies that found negative or no association in Hispanic children [59,60,61] and positive association in African American children [62]. Interestingly, 67.8% of Hispanic/Latino and 55.1% of African American parents in this study reported eating meals with their family seven times/week, which was higher than the Food Marketing Institute (FMI) estimate (50%) of U.S. families with children who eat meals together [63]. In fact, some studies found minority and low-income families to partake in fewer family mealtimes than White families [51,64]. Challenges that may limit routine family mealtimes include conflicting parent work schedules, after-school activities, lack of suitable space, and negative child behaviors, and were more prominent in low-income, African American families [65]. Future studies are needed to further examine the influence of family mealtimes on child FV intake in each racial/ethnic group independently, so that findings may provide an insight to the disparity that emerged in the current study. Additionally, research is needed to determine feasible ways in which parents can increase the frequency of family meals to improve child FV intake. 

Lastly, parents that communicated about healthy eating and nutrition with their child at least sometime in the past 6 months were associated with increased child FV intake among Hispanics/Latinos. This result concurred with a previous study demonstrating an active parent’s guidance and education to be associated with higher FV consumption among children [66]. However, because our analysis assessed the frequency of parent’s communicating about healthy eating and nutrition, our results cannot be directly compared to other studies that may have explored parent–child communication differently. 

Further investigation of the influence of SNAP participation was conducted, and interestingly, participating in SNAP moderated the association between parent and child FV intake among Hispanics/Latinos. Furthermore, the effect of SNAP participation became stronger and statistically significant as the frequency of parent FV intake increased, suggesting that SNAP participation may be less impactful on improving child FV intake if parents infrequently consume FV. These results underscore the importance of programs such as Brighter Bites that provide fresh produce for the whole family consistently. Moreover, this finding adds to the current body of literature on the effects of SNAP participation on the relationship between parent and child FV intake. More research is warranted to better understand the role of SNAP participation and parent FV consumption on improving FV consumption among children, and why the impact may be different between African American and Hispanic/Latino children that are eligible to participate in SNAP.

### 4.1. Strengths

The large sample size of low-income, minority households from assorted geographic areas within the U.S. allowed for the Hispanic/Latino and African American populations to be assessed independently, as opposed to only one minority group. In addition, this study examined the relationship between frequency of cooking meals from scratch and child FV intake, which was yet to be extensively explored in past literature. 

### 4.2. Limitations

This use of cross-sectional data in this study cannot determine causality. Fruit and vegetable intake and home nutrition environment behaviors were self-reported; leading to a potential social desirability bias and recall bias. Furthermore, self-selection bias may exist since a larger sample of Hispanic/Latino families (*n* = 5601) had enrolled in Brighter Bites and had a higher response rate to the Fall survey compared to African American families (*n* = 473). The sample size was unequal, which could bias the findings and make comparison between groups difficult. Lastly, this study used a secondary analysis conducted on data that were originally collected using a convenience sample consisting of a low-income minority population, in which generalizability of the study findings may be limited. 

## 5. Conclusions

In conclusion, our study contributes to the previous literature that found a positive relationship between parent and child FV intake. The home nutrition environment was positively associated with child FV intake, and this association varied by race/ethnicity. Our results revealed that all children benefited from frequent cooked meals from scratch at home and FV served at meals; however, only Hispanic/Latino children benefited from frequent family mealtimes and parent communication about healthy eating and nutrition. Thus, more research is needed to identify barriers specific to each racial/ethnic group and to determine effective strategies to overcome these barriers, in order to improve child FV consumption and overall nutrition equity. This study highlighted the critical demand for nutrition programs such as Brighter Bites that utilize effective strategies such as weekly produce distributions, nutrition education, and recipe tasting to improve FV intake and the home nutrition environment of low-income and minority children and parents. In addition, this study encouraged the use of culturally tailored interventions that allow the ability to address the influences that would match the child’s race, culture, and ethnicity. Future programs and interventions should consider tailoring their messaging and strategies to low-income racial/ethnic groups independently. Lastly, utilizing SNAP participation to promote child FV intake deserves further investigation. 

## Figures and Tables

**Table 1:** Demographic Characteristics of Brighter Bites Participants (*n* = 6074) Stratified by Race/Ethnicity, Fall 2018 Pre-Parent Survey.

Variable	Total (*n* = 6074)	Hispanic/Latino (*n* = 5601)	African American (*n* = 473)	*p* Value *
	_____________mean ± sd_______________			
Age (years)				
Adult	34.87 ± 7.787	34.57 ± 7.44	36.92 ± 9.24	*** <0.001
Child	6.53 ± 2.00	6.53 ± 1.99	6.34 ± 2.10	0.096
Household size (*n*)				
Adult	2.45 ± 1.09	2.48 ± 1.09	2.15 ± 1.14	*** <0.001
Child	2.62 ± 1.17	2.64 ± 1.16	2.60 ± 1.26	0.529
Total	5.05 ± 1.58	5.08 ± 1.56	4.65 ± 1.73	*** <0.001
	__n (col %)__	_______n (row %)_______		
City				*** <0.001
Austin	871 (14.34)	787 (90.36)	84 (9.64)	
Dallas	1572 (25.86)	1479 (94.14)	92 (5.86)	
Houston	3145 (51.78)	2940 (93.48)	205 (6.52)	
SW Florida	231 (3.80)	221 (95.67)	10 (4.33)	
Washington DC	256 (4.21)	174 (67.97)	82 (32.03)	
	___________________n (col %)_________________			
Child gender				0.122
Male	3019 (50.74)	2802 (51.03)	217 (47.28)	
Female	2931 (49.26)	2689 (48.97)	242 (52.72)	
Parental employment				*** <0.001
Employed (full/part time)	1721 (31.39)	1460 (28.86)	261 (61.70)	
Self-employed	312 (5.69)	282 (5.57)	30 (7.09)	
Homemaker	2944 (53.70)	2892 (57.17)	52 (12.29)	
Unemployed	400 (7.30)	347(6.86)	53 (12.53)	
Unable to work	105 (1.92)	78 (1.54)	27 (6.38)	
Language spoke at home				*** <0.001
English only	968 (16.08)	546 (9.84)	422 (89.79)	
Bilingual	2157 (35.82)	2150 (38.73)	7 (1.49)	
Spanish only	2852 (47.35)	2845 (51.25)	6 (1.28)	
Other language(s)	45 (0.75)	10 (0.18)	35 (7.45)	
Parental education				*** <0.001
Never attended school or only kindergarten	77 (1.38)	77 (1.49)	0	
Grades 1–8	1015 (18.13)	1009 (19.57)	6 (1.35)	
Grades 9–11	1172 (20.93)	1145 (22.21)	27 (6.08)	
Grade 12 or GED	1981 (35.38)	1830 (35.50)	151 (34.01)	
College 1–3 years	1005 (17.95)	851 (16.51)	154 (34.68)	
College 4 years or more (graduate)	349 (6.23)	243 (4.71)	106 (23.87)	
Food security status				* 0.046
Food insecure	4075 (71.88)	3734 (71.53)	341 (75.95)	
Food secure	1594 (28.12)	1486 (28.47)	108 (24.05)	
	_______________n (yes %)_______________			
Government assistance program				
WIC	1581 (26.84)	1470 (27.03)	111 (24.50)	0.243
SNAP	2001 (34.23)	1780 (33.04)	221 (48.15)	*** <0.001
Double Dollars	63 (1.09)	52 (0.97)	11 (2.46)	** 0.004
Medicaid	3488 (59.59)	3243 (60.03)	245 (54.32)	* 0.018
Medicare	354 (6.10)	277 (5.17)	77 (17.34)	*** <0.001
Free + reduced meals/NSLP	4315 (74.84)	3928 (74.00)	387 (84.50)	*** <0.001
CHIP	1204 (20.96)	1102 (20.78)	102 (23.08)	0.255

Abbreviations: CHIP, Children’s Health Insurance Program; SNAP, Supplemental Nutrition Assistance Program; WIC, Special Supplemental Nutrition Program for Women, Infants, and Children; GED, General Educational Development test; NSLP, National School Lunch Program.* Boldface denotes significant differences (* *p* < 0.05, ** *p* < 0.01, *** *p* < 0.001) between ethnic groups; determined by *t*-test or chi-square test.

**Table 2:** Baseline Characteristics of Dietary Fruit and Vegetable (FV) Intake and the Home Nutrition Environment of Brighter Bites Participants (*n* = 6074) Stratified by Race/Ethnicity, Fall 2018 Pre-Parent Survey.

Variable	Total (*n* = 6074)	Hispanic/ Latino (*n* = 5601)	African American (*n* = 473)	*p* Value *
	___________mean ± sd________________			
Dietary FV intake [Scale Range] (times/day)				
Child ^1^ [0–5]	2.05 ± 0.78	2.02 ± 0.76	2.34 ± 0.88	*** <0.001
Parent ^2^ [0–2]	1.28 ± 0.51	1.28 ± 0.51	1.32 ± 0.51	0.572
	_____________n (col %)________________			
*Home Nutrition Environment*				
Vegetables served at mealtimes				*** <0.001
Never	380 (6.32)	366 (6.60)	14 (2.97)	
1–2 times per week	1986 (33.02)	1877 (33.86)	109 (23.14)	
3–4 times per week	2094 (34.81)	1924 (34.70)	170 (36.09)	
5–6 times per week	843 (14.01)	764 (13.78)	79 (16.77)	
7 times per week	712 (11.84)	613 (11.06)	99 (21.02)	
Fruits served at mealtimes				** 0.001
Never	703 (11.72)	621 (11.24)	82 (17.37)	
1–2 times per week	2058 (34.32)	1900 (34.40)	158 (33.47)	
3–4 times per week	1711 (28.54)	1582 (28.64)	129 (27.33)	
5–6 times per week	771 (12.86)	725 (13.12)	46 (9.75)	
7 times per week	753 (12.56)	696 (12.60)	57 (12.08)	
Family mealtimes				*** <0.001
Never	22 (0.37)	17 (0.31)	5 (1.07)	
1–2 times per week	506 (8.55)	460 (8.44)	46 (9.83)	
3–4 times per week	736 (12.43)	642 (11.78)	94 (20.09)	
5–6 times per week	700 (11.82)	635 (11.65)	65 (13.89)	
7 times per week	3956 (66.82)	3698 (67.83)	258 (55.13)	
Parent–child communication				*** <0.001
Never	86 (1.43)	81 (1.46)	5 (1.07)	
Rarely	314 (5.23)	285 (5.15)	29 (6.18)	
Sometimes	1967 (32.77)	1855 (33.53)	112 (23.88)	
Most of the time	2040 (33.99)	1897 (34.29)	143 (30.49)	
All of the time	1595 (26.57)	1415 (25.57)	180 (38.37)	
Meals cooked from scratch				*** <0.001
Never	120 (2.10)	109 (2.08)	11 (2.36)	
Less than once a month	201 (3.52)	182 (3.47)	19 (4.08)	
Less than once a week	187 (3.27)	162 (3.09)	25 (5.36)	
Once a week	324 (5.67)	301 (5.74)	23 (4.94)	
A few times a week	1041 (18.23)	864 (16.48)	177 (37.98)	
Once a day	2274 (39.82)	2230 (42.52)	44 (9.44)	
A few times a day	1563 (27.37)	1396 (26.62)	167 (35.84)	

^1^ Child FV intake including fruit juice and fried potatoes ^2^ Parent FV intake without fruit juice and fried potatoes. * Boldface denotes significant differences (* *p* < 0.05, ** *p* < 0.01, *** *p* < 0.001) between ethnic groups; determined by *t*-test or chi-square test.

**Table 3:** Associations Between Parent Fruit and Vegetable (FV) Intake, Home Nutrition Environment and Child FV Intake Stratified by Race/Ethnicity.

Variable	Hispanic/Latino (*n* = 5601)		African American (*n* = 473)	
	Adjusted Mean Difference in Child FV Intake ß ^a^ (95% CI)	*p* Value *	Adjusted Mean Difference in Child FV Intake ß ^a^ (95% CI)	*p* Value *
Dietary intake				
Parent FV intake (times/day)	0.701 ^1^ (0.650, 0.751)	*** <0.001	0.916 ^2^ (0.762, 1.07)	*** <0.001
Home nutrition environment				
Vegetables served at mealtimes (reference: never)				
1–2 times per week	0.011 (−0.079, 0.102) ^3^	0.808	0.103 (−0.054, 0.748) ^8^	0.755
3–4 times per week	0.318 (0.228, 0.409) ^3^	*** <0.001	0.486 (−0.146, 1.12) ^8^	0.132
5–6 times per week	0.710 (0.609, 0.811) ^3^	*** <0.001	0.765 (0.107, 1.40) ^8^	* 0.022
7 times per week	0.857 (0.753, 0.962) ^3^	*** <0.001	1.09 (0.450, 1.74) ^8^	** 0.001
Fruits served at mealtimes (reference: never)				
1–2 times per week	−0.021 (−0.098, 0.056) ^4^	0.587	0.262 (0.004, 0.520) ^8^	* 0.046
3–4 times per week	0.259 (0.180, 0.337) ^4^	*** <0.001	0.443 (0.178, 0.708) ^8^	** 0.001
5–6 times per week	0.538 (0.447, 0.629) ^4^	*** <0.001	0.858 (0.520, 1.20) ^8^	*** <0.001
7 times per week	0.690 (0.598, 0.782) ^4^	*** <0.001	0.992 (0.673, 1.31) ^8^	*** <0.001
Family mealtimes (reference: never)				
1–2 times per week	0.030 (−0.395, 0.456) ^5^	0.889	−0.190 (−1.06, 0.675) ^8^	0.667
3–4 times per week	0.190 (−0.232, 0.613) ^5^	0.378	0.040 (−0.803, 0.884) ^8^	0.925
5–6 times per week	0.335 (−0.088, 0.758) ^5^	0.120	0.448 (−0.408, 1.30) ^8^	0.305
7 times per week	0.506 (0.087, 0.924 )^5^	* 0.018	0.466 (−0.366, 1.30) ^8^	0.272
Parent–child communication (reference: never)				
Rarely	0.092 (−0.134, 0.319) ^6^	0.424	−0.306 (−1.30, 0.685) ^8^	0.545
Sometimes	0.266 (0.063, 0.468) ^6^	* 0.010	0.136 (−0.806, 1.08) ^8^	0.778
Most of the time	0.482 (0.279, 0.684) ^6^	*** <0.001	0.319 (−0.618, 1.26) ^8^	0.505
All of the time	0.609 (0.405, 0.812) ^6^	*** <0.001	0.544 (−0.391, 1.48) ^8^	0.254
Meals cooked from scratch (reference: never)				
Less than once a week	−0.162 (−0.333, 0.008) ^7^	0.062	0.474 (−0.035, 0.983) ^9^	0.068
Once a week	−0.016 (−0.163, 0.131) ^7^	0.834	0.175 (−0.348, 0.698) ^9^	0.512
A few times a week	−0.064 (−0.189, 0.061) ^7^	0.314	0.132 (−0.233, 0.497) ^9^	0.478
Once a day	0.049 (−0.065, 0.164) ^7^	0.398	0.388 (−0.062, 0.839) ^9^	0.091
A few times a day	0.144 (0.026, 0.262 )^7^	* 0.017	0.510 (0.140, 0.881) ^9^	** 0.007

Abbreviations: Cl, Confidence interval. ^a^ Regression coefficients were calculated using Multilevel Mixed Effects Linear Regression models. * Boldface indicates statistical significance at * *p* < 0.05, ** *p* < 0.01, *** *p* < 0.001 ^1^ Adjusted for SNAP, Medicare, parent age, language spoken at home, child age, city, food insecurity status and interaction between parent FV intake and SNAP (*p* = 0.020). ^2^ Adjusted for SNAP, child age, parent education, and food insecurity status. ^3^ Adjusted for SNAP, Medicare, parent age, language spoken at home, total household size, child age, city, and food insecurity status. ^4^ Adjusted for SNAP, Medicare, parent age, language spoken at home, total household size, child age, and food insecurity status. ^5^ Adjusted for SNAP, Medicare, language spoken at home, total household size, child age, and food insecurity status. ^6^ Adjusted for SNAP, Medicare, parent age, language spoken at home, total household size, child age, NSLP, and food insecurity status. ^7^ Adjusted for SNAP, Medicare, language spoken at home, total household size, child age, and food insecurity status. ^8^ Adjusted for child age, parent education, and food insecurity status. ^9^ Adjusted for SNAP, child age, parent education, and food insecurity status.

## Data Availability

Data are unavailable due to privacy or ethical restrictions.

## Supplementary Materials

The following supporting information can be downloaded at: https://www.mdpi.com/article/10.3390/nu15081819/s1, Table S1: Association Between SNAP Participation and Child FV Intake at each Level of Parent FV Intake Frequency Among Hispanic/Latinos (Interaction Effect between SNAP Participation and Parent FV Intake in relation to Child FV Intake). Figure S1: Contrasts of Predictive Margins: SNAP by Hispanic/Latino Parent FV Intake with 95% Confidence Intervals. Text S1: Response Categories/Options of Variables.
