# Supplementary material for: Fruit and Vegetable Intake and Home Nutrition Environment among Low-Income Minority Households with Elementary-Aged Children
Source: Nutrients. 2023 Apr 10;15(8):1819. doi: 10.3390/nu15081819 (PMC10145522; doi:10.3390/nu15081819)
Supplement: Supplementary file 1 [file nutrients-15-01819-s001.zip › nutrients-2252822-supplementary.pdf]

Supplemental Table S1: Association Between SNAP participation and Child FV Intake at each Level of Parent FV Intake Frequency Among Hispanic/Latinos (Interaction Effect Between SNAP Participation and Parent FV Intake in relation to Child FV Intake)

Parent FV Intake	Adjusted Mean Difference in Child FV Intake between SNAP vs. no-SNAP ß[a] (95% CI)	P value*
0 times/day (n = 11)	-0.046 (-0.160, 0.068)	0.430
0.214 times/day (n = 49)	-0.031 (-0.133, 0.070)	0.545
0.429 times/day (n = 499)	-0.017 (-0.107, 0.073)	0.712
0.5 times/day (n = 33)	-0.002 (-0.081, 0.076)	0.951
0.714 times/day (n = 607)	0.012 (-0.056, 0.080)	0.728
0.786 times/day (n = 20)	0.026 (-0.032, 0.085)	0.373
1 times/day (n = 1,091)	0.041 (-0.010, 0.091)	0.113
1.214 times/day (n = 91)	0.055 (0.010, 0.101)	***0.018**
1.286 times/day (n = 733)	0.070 (0.025, 0.115)	****0.002**
1.5 times/day (n = 309)	0.084 (0.036, 0.132)	****0.001**
1.571 times/day (n = 660)	0.099 (0.045, 0.153)	*****<0.001**
1.786 times/day (n = 538)	0.113 (0.051, 0.176)	*****<0.001**
2 times/day (n = 911)	0.128 (0.055, 2.002)	*****<0.001**

Abbreviations: FV, fruit and vegetable; CI, confidence interval.

[*]Boldface indicates statistical significance at *P<0.05, **P<0.01, ***P<0.001

[a]Adjusted for SNAP, Medicare, parent age, language spoken at home, child age, city, and food insecurity status.

Figure S1. Contrasts of Predictive Margins: SNAP by Hispanic/Latino Parent FV Intake with 95% Confidence Intervals

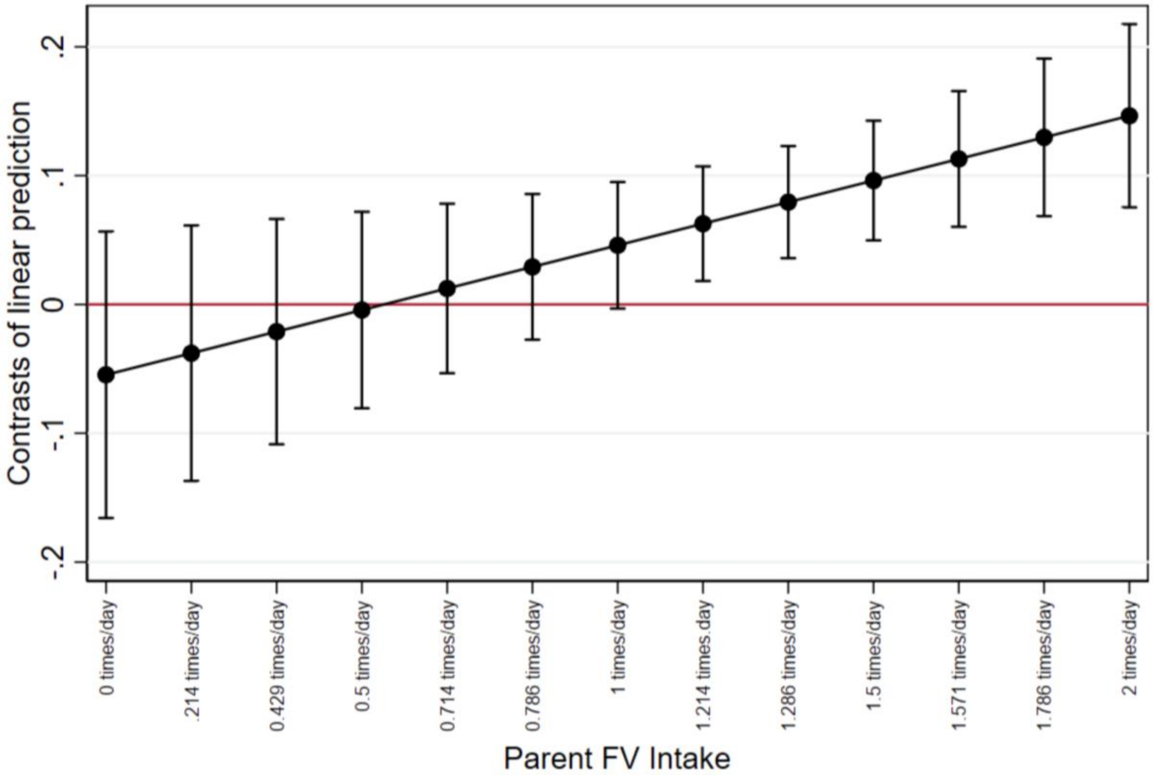

Text S1: Response Categories/Options of Variables

Child FV intake
During the past 7 days, how many times did your child:
1. Drink 100% pure fruit juice like orange, apple, grape, etc.?
2. Eat fruit per day?
3. Eat a green salad or non-friend vegetables like carrots, broccoli, collards, green beans, corn, etc.?
4. Eat fried potatoes like French fries, tater tots, hash brown potatoes, etc.?
5. Eat any other kind of potatoes for example, how many times did you eat potatoes that were baked, boiled, mashed, used in soups or stews, etc.?

Responses:
- Never
- 1-2 times per week
- 3-4 times per week
- 5-6 times per week
- 7 times per week

Parent FV intake
During the past 7 days, how many times:
1. Did you eat fruits like apples, bananas, melons, etc.?
2. Did you eat vegetables like greens, carrots, peppers, tomatoes, squash, broccoli, cabbage, cauliflower, cucumbers, green beans, celery, sweet potatoes, etc.?

Responses:
- Never
- 1-2 times per week
- 3-4 times per week
- 5-6 times per week
- 7 times per week

Governmental Assistance Programs (Response Options: yes/no)
Does your family use the following?
- Special Supplemental Nutrition Program for Women, Infants, and Children (WIC)
- Supplemental Nutrition Assistance Program (SNAP)
- Double Dollars Incentive Program
- Medicaid/Texas Health Steps
- Medicare
- National School Lunch and/or Breakfast Programs (NSLP)/ Free/Reduced meals at school
- Children’s Health Insurance Program (CHIP)
